# Use of Long-Acting Injectable Antipsychotic in an Inpatient Unit of a Community Teaching Hospital

**DOI:** 10.1155/2019/8629030

**Published:** 2019-06-13

**Authors:** Olaniyi Olayinka, Ayotomide Oyelakin, Karthik Cherukupally, Inderpreet Virk, Chiedozie Ojimba, Susmita Khadka, Alexander Maksymenko, Patrice Fouron, Taher Khandaker, Tolu Olupona, Jason Hershberger

**Affiliations:** Department of Psychiatry and Behavioral Sciences, Interfaith Medical Center, Brooklyn, NY, USA

## Abstract

**Background:**

Individuals with Schizophrenia Spectrum Disorders (SSD) often experience significant impairment in educational, occupational, and psychosocial functioning. The clinical benefit of long-acting injectable antipsychotics (LAIs) in the management of patients with SSD is well established. SSD patients who are nonadherent to treatment have lower disease relapse and readmission rates when prescribed a LAI, compared to oral antipsychotics. Despite the reported advantages of LAIs, their prescription rates in clinical settings remain low. This pilot study aimed to determine the pattern of LAI prescription in psychiatric inpatients of a teaching community hospital in Brooklyn, New York.

**Methods:**

A retrospective review of the charts of patients discharged from the psychiatric units of the hospital from September 1, 2017, through September 30, 2017, was conducted. Frequencies and proportions for demographic and disease-related characteristics were calculated. Pertinent continuous variables were recoded into categorical variables. Chi-square-tests or Fisher's exact tests were performed for categorical variables. The one-sample Shapiro-Wilk test (for sample size < 50) was used to check for the normality of distribution of continuous variables. Statistical significance was defined as p ≤ 0.05.

**Results:**

Forty-three (70%) of the patients discharged from the inpatient unit during the study period had SSD and were eligible for a LAI. Their ages ranged from 20 to 71 years (mean = 41 years), and more than two-thirds were male. Less than half of the eligible patients (n = 19; 44%) were prescribed a LAI, most of whom were male (n=16; 84%). An association between age group (patients aged 41 years or younger) and LAI use was observed (p < 0.05), while gender, employment status, living arrangement, length of hospital stay, recent hospitalization, and cooccurring substance use disorder were not.

**Conclusion:**

LAI prescription rate at the inpatient psychiatric unit of the hospital was marginally higher than those reported in most studies. Age appears to influence LAI use during the study period. Initiatives that increase LAI prescription rate for all eligible patients admitted to inpatient psychiatric unit should be encouraged.

## 1. Introduction

Schizophrenia Spectrum Disorders (SSD) include a group of persistent, unrelenting, debilitating psychotic illnesses that cause significant impairment in educational, occupational, and psychosocial functioning of sufferers. The clinical benefits of antipsychotic agents, particularly long-acting injectable antipsychotics (hereinafter referred to as LAI), in the management of patients with schizophrenia spectrum disorders are well established. Recent studies show a lower rate of disease relapse, a decrease in readmission rates, and an increase in medication adherence among schizophrenia patients prescribed LAIs, compared with oral antipsychotics [1–3]. For example, a 2011 Finnish study involving approximately 2,600 patients with schizophrenia found that the risk of readmission was about 33% of that for patients receiving oral antipsychotics [3]. This finding is in keeping with similar studies conducted in other parts of the world [4–6]. Of note, a recent study conducted in the United States showed a reduction in treatment failure, police arrest, and incarceration among patients who received a novel LAI, compared with oral medications [7].

Given the aforementioned benefits, several guidelines have emerged recommending the use of LAI antipsychotics for treatment of chronic psychotic disorders [8, 9]. Despite these recommendations, as well as the easy availability of depot formulations, the use of LAIs in the United States and many parts of the world remains low [4, 5, 10]. For example, recent epidemiologic studies found that the prescription rates of LAI in clinical settings vary between 10% and 33% [4, 5, 8]. Additionally, psychiatrists appear to have a conservative attitude towards LAIs. This pilot study aimed to determine the pattern of LAI prescription in psychiatric inpatients of a teaching community hospital in Brooklyn, New York.

## 2. Methods

### 2.1. Study Design, Setting, and Population

This was a retrospective review of the charts of patients discharged from the psychiatric unit of Interfaith Medical Center from September 1, 2017, through September 30, 2017.

The hospital is a community teaching facility based in Central Brooklyn, New York City, mainly serving low-income, underserved, and uninsured patient population. The facility has a 90-bed inpatient psychiatric unit, psychiatric emergency services, 40-bed detoxification/rehabilitation units, and various outpatient behavioral health services.

The inclusion criteria for this study were (1) all patients diagnosed with SSD prior to discharge, (2) those aged 18 years and older, and (3) those eligible for a LAI; per the policy of the hospital, LAIs are recommended to patients: (I) with a history of multiple or frequent hospitalizations, (II) with a history of poor adherence with oral antipsychotic medications, and (III) based on personal preference. Because the indication for administering a LAI was difficult to ascertain from the charts, all patients with SSD were considered eligible for a LAI in this study.

### 2.2. Sampling

All patient discharged from the psychiatric unit in the month of September, 2017, were included in the study.

### 2.3. Data Collection, Management, and Analysis

Extracted data were entered into a medical chart abstraction form designed by the study team, checked for completeness, and exported into SPSS version 20 (IBM, Armonk, NY, USA) for analysis. Frequencies and proportions for demographic and disease-related characteristics were calculated. Pertinent continuous variables were recoded into categorical variables as follows: age was recoded into two age categories based on the mean age of patients of 41 years which followed a normal distribution (i.e., aged 41 years and younger = 1; patients aged 42 years or older = 2; number of rehospitalization was recoded as “1” for no rehospitalization within 30 days prior to current admission and “2” for patients with at least one hospitalization within 30 days of current admission; length of hospital stay (LOS) was recoded as “1” for those discharged by day 13 of hospital stay and “2” for those with hospital stay longer than 13 days; 13 days was chosen based on the median LOS of patients. Median LOS, as opposed to mean LOS, was used as cut-off because of the skewed nature of the data (LOS ranged from 2 days to 42 days).

Cooccurring substance use disorder in patients with SSD was determined using the Diagnostic and Statistical Manual of Mental Disorders, 5th Edition (DSM-5) criteria; i.e., patients must meet at least two of the eleven DSM-5 drug use disorder criteria within 12 months prior to current hospitalization. Missing data were coded as “9999” and excluded from analysis.

Chi-square-tests or Fisher's exact tests were performed for categorical variables. The one-sample Shapiro-Wilk test (for sample size < 50) was used to check the normality of distribution of continuous variables. Statistical significance was defined as p ≤ 0.05.

### 2.4. Ethical Consideration

Institutional review board approval was obtained for this study.

## 3. Results

Sixty-one patients were discharged from the inpatient unit during the study period. Of these, 43 patients (70%) were hospitalized for SSD and were eligible for a LAI (Table 1). Regarding those with SSD (n=43), their ages ranged from 20 to 71 years (mean = 41 years), and most were male (n=33; 77%). There was no significant difference in the demographic characteristic of all the patients (n=61), compared to those diagnosed with SSD (n = 43). Less than half of eligible patients (n = 19; 44%) were prescribed a LAI (i.e., Risperdal Consta [47%], Invega Sustenna [42%], and Haldol Decanoate [11%]) most of whom were male (n=16; 84%). Additionally, all were unemployed and rehospitalized at least once within 30 days of discharge while over half of the patients (n=10; 53%) were homeless. The prevalence of tobacco, cannabis, alcohol, and cocaine use disorders in those who received a LAI was 65% (n=11), 39% (n=7), 24% (n=4), and 6% (n=1), respectively (Table 1).

A statistically significant association between patients' age group (i.e., those aged 41 years or younger) and LAI administration was observed, *χ*2(1) = 4.408, p = 0.036, while gender, *χ*2(1) = 1.063, p = 0.302, employment status, *χ*2(1) = 1.661, p = 0.198, living arrangement, *χ*2(1) = 1.504, p = 0.2201, number of rehospitalizations, *χ*2(1) = 1.773, p = 0.183, as well as cooccurring tobacco use disorder, *χ*2(1) = 0.001, p = 0.977, cannabis use disorder, *χ*2(1) = 0.744, p = 0.388, and alcohol use disorder, *χ*2(1) = 0.135, p = 0.714, were not. Approximately 44% (n=19) of patients with length of hospital stay greater than 13 days were prescribed a LAI; however, this association was not statistically significantly, *χ*2(1) = 0.985, p = 0.321.

## 4. Discussion

Long-acting injectables have proven to be more efficacious in preventing rehospitalization and improving medication adherence among patients with schizophrenia, compared to oral antipsychotics [11, 12]. Recent studies have also shown them to be effective in the treatment of acute or first episode psychosis [10]. LAI, though efficacious, is found to be less utilized in clinical practice with available studies reporting prevalence of LAI use of approximately 30 percent or less [4, 12]. For example, a 2009 study of over 1500 patients with schizophrenia hospitalized in forensic units in the United Kingdom found that 28% of the patients were prescribed a LAI antipsychotic [4]. Similarly, while reviewing the extant literature to inform guidelines for LAI use in France, the authors found that LAI prescription rates are generally low across Europe; the United Kingdom reportedly had the highest LAI prescription rate of 29 percent [8]. A higher prevalence of LAI use, however, has been reported in few clinical settings. A 16-month study conducted in a major outpatient clinic in Hong Kong found that approximately 40 percent of 270 patients with clinically stable schizophrenia seen at the clinic received a LAI [6]. The findings of the Hong Kong study are similar to what we found in our pilot study; that is, 44% of eligible patients were prescribed a LAI.

Few factors have been hypothesized to be responsible for the relatively high use of LAI antipsychotics in our study. These include the attitude, knowledge, and experience of health providers regarding LAIs [4, 5, 13, 14]. For example, Waddell and Taylor found in their analysis of 12 articles on LAI use that, although patients' attitudes towards LAI are generally negative, a sizeable proportion of patients showed preference for LAI antipsychotics; patients' preference was noted to increase with experience of LAIs [14]. Similarly, the authors found that older age as well as a positive attitude and knowledge regarding the side effects of LAIs correlated positively with psychiatrists' use of LAIs.

Many factors such as sociodemographic characteristics, previous noncompliance with medication, and past side effects to antipsychotics impact the use of LAI [4, 5]. For instance, a positive correlation between younger age and LAI prescription has been reported in some studies, while others found no statistically significant differences in LAI use by age [15, 16]. We found patients who are aged 41 years or less to be significantly associated with LAI use in our unit during the study period. A similar relationship was reported by Soleman and colleagues, following their recent analysis of the medical records of over 63,000 inpatients in Los Angeles county [15]. The authors found that patients aged 34 years or younger were more likely to be prescribed a LAI, compared to their corresponding oral formulation. The increasing recognition of the effectiveness of second-generation LAIs in treating first episode of psychosis, the authors posit, might explain the trend in LAI prescription among young patients. The higher rates of nonadherence with antipsychotics among younger patients may also increase the use of LAIs in this age group [17, 18].

Anecdotal information obtained from a senior inpatient psychiatrist at our hospital suggests that the increased use of LAI in younger patients might reflect the effort to prevent future relapse and subsequent readmission in this age group that have a higher prevalence of cooccurring substance use disorders. Pharmacologically, the preference for LAIs over oral antipsychotics might be related to their pharmacokinetic, injectable antipsychotics bypass hepatic first pass metabolism, hence increasing their bioavailability [12]. Exploiting this pharmacologic advantage is particularly crucial in the management of SSD patients with cooccurring tobacco use disorder. Specifically, chronic nicotine exposure may (1) precipitate frequent relapse in patients with SSD on clozapine augmentation or monotherapy by increasing the metabolism of clozapine via induction of the cytochrome P450 1A2 subenzyme and (2) indirectly promote medication nonadherence as studies have shown that it attenuates the cognitive impairment associated with the use of antipsychotics including clozapine, haloperidol, and risperidone [19, 20]. Of note, report of the negative impact of smoking on other antipsychotics is emerging [21].

Patients' responses when offered LAIs have been more neutral than unfavorable when the discussions are made in a timely manner in inpatient settings [22]. The use of LAI is also impacted by the high cost of the medication particularly for underinsured/uninsured patients, hence making the drug inaccessible to the patients that need it [10].

## 5. Limitation and Strength of the Study

The results of this study are subject to several limitations. This is a retrospective study; hence, the quality of the data abstracted depends on proper chart documentation. The low sample size might have impacted the ability of this study to detect small differences between variables and LAI prescription. Additionally, this study did not evaluate the impact of use of the different LAIs. This is significant because studies have shown that newer LAI antipsychotics are better tolerated than the first-generation antipsychotics and have lesser extrapyramidal side effects. Second generation antipsychotics such as Risperidone are however known for their metabolic side effects, which affect its use in patients who have coexisting metabolic conditions [12]. The study also did not explore whether patients presented with first episode of schizophrenia, if the LAI administered was the first antipsychotic treatment, and the LAI treatment preferences of patients. This is because this information could not be retrieved from patients' charts.

## 6. Conclusion

Although LAI has been of advantage in the treatment of schizophrenia and other psychotic illnesses, it is being underutilized. Clinicians misperceptions about patient acceptance and their pattern of practice have been found to impact its use. There is a need for increased awareness about the risk of side effects and the benefits of its use as evidenced by current guidelines on schizophrenia management. Regarding our center, the study will be expanded to include all psychiatric inpatient units, pattern of use for different LAI, and study timeline expanded. The goal will be to continue to offer LAI to patients to facilitate treatment, resolution of symptoms, and improved outcomes.

## Figures and Tables

**Table 1:** Demographic information of study subjects.

Characteristic	All Patients	Patients with SSD	Patients who received a LAI (n = 19)
	(n = 61)	(n = 43)	
Gender (%)			
(i) Female	18 (30)	10 (23)	3 (16)
(ii) Male	43 (70)	33 (77)	16 (84)

Mean age, years (range)	41 (20—71)	41 (20—71)	39 (20—71)

Race (%)			
(i) Black	49 (80)	35 (81)	17 (89)
(ii) White	11 (18)	8 (19)	2 (11)
(iii) Others	1 (2)	0 (0)	0 (0)

Unemployed (%)	56 (91)	41 (95)	19 (100)

Readmission within 30 days of last discharge	25 (41)	20 (47)	11 (58)

Homeless	32 (52)	26 (60)	10 (53)

Average length of stay, days	14	16	18
Median length of stay, days	11	13	14

*∗*Co-occurring substance use disorder (%)			
(i) Tobacco	38/56 (68)	25/39 (64)	11/17 (65)
(ii) Cannabis	26/55 (47)	19/40 (48)	7/18 (39)
(iii) Alcohol	14/55 (25)	8/39 (21)	4/17 (24)
(iv) Cocaine	8/55 (15)	4/40 (10)	1/18 (6)
(v) Synthetic Cannabinoids	2/55 (4)	2/40 (5)	1/18 (6)
(vi) Amphetamine	1/55 (2)	1/40 (3)	0/18 (0)
(vii) Any substance	49/58 (84)	33/41 (80)	13/18 (68)

*∗*Proportions calculated after missing values were excluded.

## Data Availability

The corresponding author has access to all data reported in this study and data are available upon reasonable request with permission of the Department of Psychiatry and Behavioral Sciences, Interfaith Medical Center, Brooklyn, New York.
